# Lentinan exerts synergistic apoptotic effects with paclitaxel in A549 cells *via* activating ROS-TXNIP-NLRP3 inflammasome

**DOI:** 10.1111/jcmm.12570

**Published:** 2015-04-09

**Authors:** Wei Liu, Jun Gu, Jun Qi, Xiao-Ning Zeng, Juan Ji, Zheng-Zhen Chen, Xiu-Lan Sun

**Affiliations:** a Jiangsu Key Laboratory of Neurodegeneration, Department of Pharmacology, Nanjing Medical UniversityNanjing, Jiangsu, China; b Department of Respiratory Medicine, The First Affiliated Hospital of Nanjing Medical UniversityNanjing, China

**Keywords:** paclitaxel, lentinan, ROS, NLRP3, TXNIP

## Abstract

Paclitaxel is generally used to treat cancers in clinic as an inhibitor of cell division. However, the acquired resistance in tumours limits its clinical efficacy. Therefore, the aim of this study was to detect whether co-treatment with lentinan enhanced the anti-cancer effects of paclitaxel in A549 cells. We found that the combination of paclitaxel and lentinan resulted in a significantly stronger inhibition on A549 cell proliferation than paclitaxel treatment alone. Co-treatment with paclitaxel and lentinan enhanced cell apoptosis rate by inducing caspase-3 activation. Furthermore, co-treatment with paclitaxel and lentinan significantly triggered reactive oxygen species (ROS) production, and increased thioredoxin-interacting protein (TXNIP) expression. Moreover, co-treatment with paclitaxel and lentinan enhanced TXNIP-NLRP3 interaction, and activated NLRP3 inflammasome whereat interleukin-1β levels were increased and cell apoptosis was induced. In addition, combination of paclitaxel and lentinan could activate apoptosis signal regulating kinase-1 (ASK1)/p38 mitogen-activated protein kinase (MAPK) signal which also contributed to cell apoptosis. Taken together, co-treatment with paclitaxel and lentinan exerts synergistic apoptotic effects in A549 cells through inducing ROS production, and activating NLRP3 inflammasome and ASK1/p38 MAPK signal pathway.

## Introduction

Lung cancer is one of the most common malignancies in the world. Although novel chemotherapeutic strategies against lung cancer have been developed, the recurrence as a result of chemotherapy failure or acquired resistance remains a major challenge in the clinical setting 1,2. Therefore, new drugs and new therapeutic strategies are required to enhance therapeutic efficacy.

Paclitaxel is generally used to treat patients with lung, ovarian, breast, and head and neck cancers in clinic 3–5. By binding to the β-subunit of the tubulin heterodimer, and accelerating the polymerization of tubulin, paclitaxel results in the stabilization of microtubules and inhibition of depolarization 6–8. Thus, paclitaxel can inhibit the cell division process between prophase and anaphase and eventually leads to apoptosis. Unfortunately, the clinical efficacy of paclitaxel is limited because some tumours show resistance or become resistant to it after repeated cycles of paclitaxel-based chemotherapy 9,10.

Natural compounds containing fungal β-glucans have been used to improve general health for thousands of years in China and Japan. Lentinan is one of the active ingredients purified from Shiitake mushrooms and has been approved as a biological response modifier for the treatment of gastric cancer in Japan 11,12. It has been revealed that letinan has great potential as an anti-inflammatory, anti-septic or anti-cancer drug 13,14. Recent clinical studies showed that lentinan, which is used as chemo-immunotherapy, could prolong the survival of patients with advanced gastric cancer 12. Therefore, this study aims to investigate whether co-treatment with paclitaxel and lentinan exerted the synergistic anti-cancer effects in A549 cells, and explore the involved mechanisms in the combined treatment.

## Materials and methods

### Materials

The cell culture medium RPMI-1640, foetal bovine serum (FBS), and antibiotics (penicillin/streptomycin) were purchased from Gibco-BRL (Rockville, MD, USA). Fluorometric assay kit of caspase-3 activity was supplied by BioVision (Mountain View, CA, USA). 20,70-dichlorodihydrofluorescein diacetate, acetyl ester (H_2_DCF-DA) were purchased from Invitrogen. Anti-phospho-apoptosis signal regulating kinase (ASK), anti-ASK, anti-phospho-p38 mitogen-activated protein kinase (MAPK), anti-MAPK, anti-thioredoxin-interacting protein (TXNIP) and anti-β-actin antibodies were acquired from Santa Cruz Biotechnology (Santa Cruz, CA, USA). Anti-pro-caspase 1, anti-caspase 1, anti-NLRP3, anti-pro–interleukin (IL)-1β and anti-IL-1β antibodies were supplied by Cell Signaling Technology (Beverly, MA, USA). All other chemicals were supplied by Sigma-Aldrich (St. Louis, MO, USA).

### Cell culture

The human lung cancer cell lines A549 (The Cell Bank of Type Culture Collection of Chinese Academy of Sciences, Shanghai, China) were cultured in RPMI 1640 medium supplemented with 10% FBS (Gibco-BRL, Carlsbad, CA, USA), 100 U/ml penicillin and 100 mg/ml streptomycin and were grown in an incubator with 5% CO_2_ at 37°C.

### MTT assay

Exponentially growing A549 cells were planted into 96-well plates, and treated with serial concentrations of paclitaxel or/and lentinan for 24, 48 or 72 hrs after adhesion. Cell proliferation was determined by the addition of 1 mg/ml MTT-containing medium for 4 hrs, addition of 100 μl DMSO to solubilize the formazan, and shaking for 10 min. in the dark. The absorbance at 570 nm was recorded using a multilabel counter ((Tecan Austria GmbH, Grödig, Austria).

### Western blot analysis

Cells were lysed in the lysis buffer, and the proteins of the lysates were quantified using a BCA protein assay kit (Pierce, Rockford, IL, USA). About 30 μg of total proteins was subjected to SDS-PAGE, transferred onto nitrocellulose membranes, and then blocked with 5% non-fat milk in TBST (20 mM Tris, 500 mM NaCl, and 0.1% Tween-20) at room temperature for 2 hrs with rocking. The membranes were probed with specific primary antibodies overnight at 4°C. After washing with TBST three times for 15 min. each, the membranes were incubated with horseradish-peroxidase-conjugated secondary antibodies (Santa Cruz Biotechnology Inc.) in TBST at room temperature for 1 hr, and specific protein bands were visualized using an enhanced chemiluminescence western blot detection kit. Equal protein loading was verified through rehybridization of the membranes and reprobing with anti-β-actin antibody.

### Determination of reactive oxygen species

H_2_DCF-DA, the reactive oxygen species (ROS)-specific fluorescent probe, was used to determine the intracellular ROS levels. A549 cells were loaded for 30 min. with 20 μM H_2_DCF-DA, and then washed twice with PBS. The level of fluorescence was assessed by a fluorescence plate reader (excitation wavelength: 485 nm; emission wavelength: 538 nm).

### Measurement of caspase-3 activity

Cells were lysed in the lysis buffer, and then the supernatants were subjected to the measurement of caspase-3 activity according to the protocol of Caspase-3/CPP32 Fluorometric Assay Kit (BioVision Incorporated). The fluorometric assay utilized a synthetic caspase-3 substrate consisting of the cleavage sequence for caspase-3 labelled with 7-amino-4-trifluoromethyl coumarin (AFC). Activated caspases in apoptotic cells cleaved the synthetic substrates to release the AFC, which was then quantified using an Optima Fluorescence Plate Reader (excitation wavelength: 400 nm; emission wavelength: 505 nm). Comparison of fluorescence of AFC from the co-treated groups with the control groups allows determination of the fold increase in caspase-3 activity.

### Hoechst 33342 staining

Cells were cultured on coverslips until reaching 70% confluence, then fixed in 4% formaldehyde, permeabilized in 0.5% Triton X-100. Nuclei were stained with Hoechst 33342 (Molecular Probes, EugSpring, USA) stain. Digital images were taken using an Olympus BX61 fluorescence microscope (Japan) equipped with ColorView CCD camera and analysis software.

### Statistical analysis

All data were shown as mean ± SEM. The quantitative assessments were performed in a blinded manner. The statistical difference among the groups was determined by one-way or two-way anova followed by Student-Newman–Keuls tests. *P* < 0.05 was considered to be statistically significant.

## Results

### Paclitaxel or letinan treatment alone inhibits cell proliferation in a concentration- and time-dependent manner

**Figure 1:**
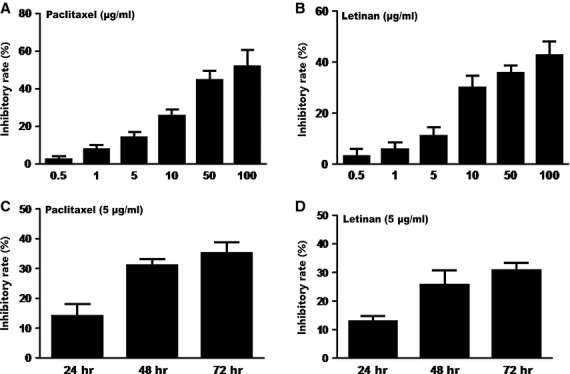
Effects of paclitaxel or letinan alone treatment on A549 cell proliferation. (A) Different concentrations of paclitaxel (0.5, 1, 5, 10, 50 and 100 μg/ml) for 24 hrs. (B) Different concentrations of letinan (0.5, 1, 5, 10, 50 and 100 μg/ml) for 24 hrs. (C) Paclitaxel alone (5 μg/ml) treated for 72 hrs. (D) Letinan alone (5 μg/ml) treated for 72 hrs. Data represent the mean ± SEM (*n* = 4).

To determine anti-proliferation effects of the individual compounds, A549 cells were treated with different concentrations of paclitaxel or letinan (0.5, 1, 5, 10, 50 and 100 μg/ml) for 24 hrs. As shown in Figure1A and B, Paclitaxel or letinan inhibited cell proliferation in a concentration-dependent manner. Paclitaxel or letinan treatment at concentrations ranging from 0.5 to 100 μg/ml decreased proliferation rates from 3.5% to 46.7%, and from 3.3% to 43.3%, respectively. As shown in Figure1C and D, paclitaxel or letinan treatment at the concentration of 5 μg/ml inhibited cell proliferation in a time-dependent manner. Paclitaxel treatment alone for 24, 48 and 72 hrs inhibited cell survival rate by 14.3%, 31.4% and 36.4%, respectively. Letinan treatment alone for 24, 48 and 72 hrs inhibited cell survival rate by 13.8%, 26.9% and 47.7%, respectively.

### Co-treatment with paclitaxel and letinan exerted synergistic inhibitory effects on A549 cell proliferation

**Figure 2:**
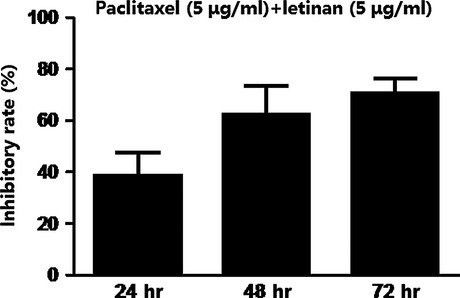
Effects of the combination of paclitaxel and letinan on A549 cell proliferation. Data represent the mean ± SEM (*n* = 4).

To investigate whether letinan could enhance the anti-cancer effects of paclitaxel, A549 cells were incubated with 5 μg/ml paclitaxel and 5 μg/ml letinan for 24, 48 and 72 hrs. The results showed that co-treatment with paclitaxel and letinan for 24, 48 and 72 hrs decreased cell survival by 38%, 62% and 72%, respectively. Our data revealed that the inhibitory effects induced by the co-treatment of paclitaxel and letinan were stronger than that of paclitaxel or letinan treatment alone, which suggested that combination of paclitaxel and letinan had synergistic effects on the inhibition of A549 cell proliferation (Fig.2).

### Co-treatment with paclitaxel and letinan triggers ROS over-production

**Figure 3:**
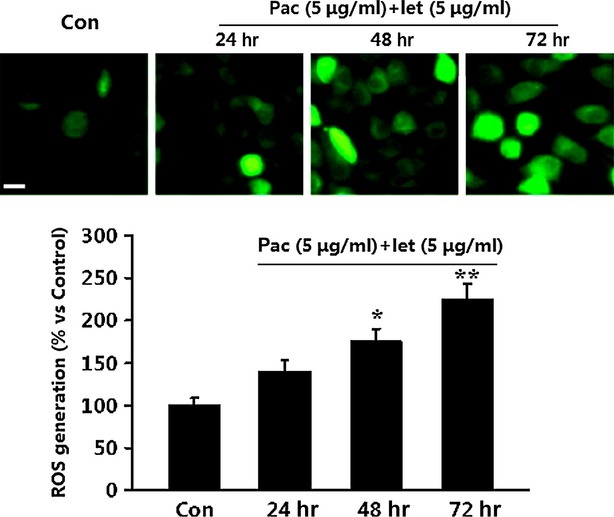
Effects of the combination of paclitaxel and letinan on ROS production in A549 cells. Data represent the mean ± SEM, *n* = 4. *P < 0.05, **P < 0.01 *versus* Con group. Con: control group; pac: paclitaxel; let: letinan.

Reactive oxygen species is an initiator of the apoptotic response in anti-cancer effects of drugs. So we investigated whether ROS generation was involved in co-treatment of paclitaxel and letinan-induced cell death. As shown in Figure3, DCF fluorescence, indicated the intracellular ROS levels, was increased gradually in A549 cells after combined treatment. Co-treatment with paclitaxel and letinan significantly induced ROS production by 1.7 (48 hrs) - and 2.2 (72 hrs) - fold, respectively.

### Co-treatment with paclitaxel and letinan induces phosphorylations of ASK1 and p38 MAPK

**Figure 4:**
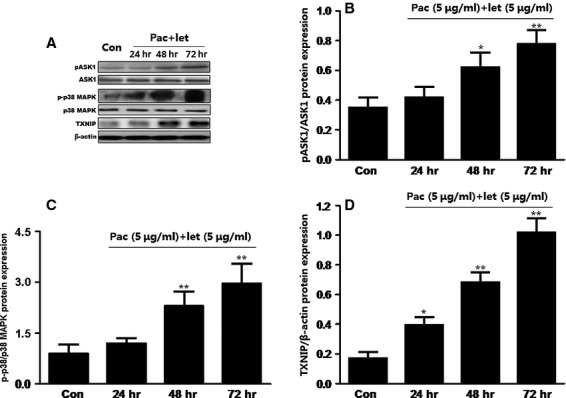
Effects of the combination of paclitaxel and letinan on the expressions of p-ASK1 (B), p-p38 MAPK (C) and TXNIP (D). Data represent the mean ± SEM, *n* = 3. *P < 0.05, **P < 0.01 *versus* Con group. Con: control group; pac: paclitaxel; let: letinan.

Reactive oxygen species acts as a mediator of ASK1, a redox-sensitive mitogen-activated protein kinase kinase kinase, which can increase p38 MAPK protein expression and activity and results in the inhibition of cell proliferation. However, it is unclear whether co-treatment with paclitaxel and letinan modulates ASK1 phosphorylation and p38 MAPK activity in A549 cells. We found that co-treatment with paclitaxel and letinan significantly potentiated the activations of ASK1 and p38 MAPK in A549 cells. Treated with paclitaxel and letinan for 24, 48 and 72 hrs, ASK1 phosphorylation was increased to 0.42 ± 0.07, 0.62 ± 0.1 and 0.78 ± 0.09, respectively. Phosphorylation of p38 MAPK was also time-dependently increased (Fig.4A–C).

### Co-treatment with paclitaxel and letinan enhances TXNIP expression and TXNIP-NLRP3 interaction

**Figure 5:**
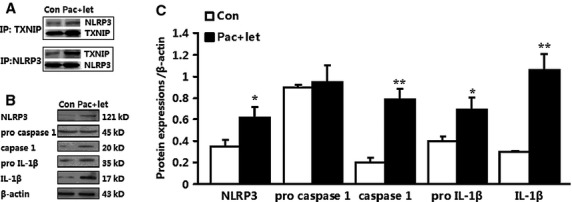
Effects of the combination of paclitaxel and letinan on TXNIP-NLRP3 interaction (A) and NLRP3 inflammasome activation (B and C). Data represent the mean ± SEM, *n* = 3. *P < 0.05, **P < 0.01 *versus* Con group. Con: control group; pac: paclitaxel; let: letinan.

Thioredoxin-interacting protein has been shown to be an effective link between increased oxidative stress and activation of the innate immune response mediated by the NLRP3 inflammasome. In this study, we found that co-treatment with paclitaxel and letinan could significantly up-regulate TXNIP expression in a time-dependent manner (Fig.4A and D). Importantly, inflammasome activators can induce the dissociation of TXNIP from thioredoxin in a ROS-sensitive manner and the binding of TXNIP to NLRP3. Our results revealed that combined treatment with paclitaxel and letinan for 72 hrs enhanced TXNIP interaction with NLRP3. Consequently, the expressions of cleaved caspase-1 and mature IL-1β, the end products of inflammasome activation, were also higher in the combined treatment groups compared to paclitaxel or letinan treatment alone (Fig.5).

### Co-treatment with paclitaxel and letinan promotes caspase-3 activation, thereby enhancing A549 cell apoptosis

**Figure 6:**
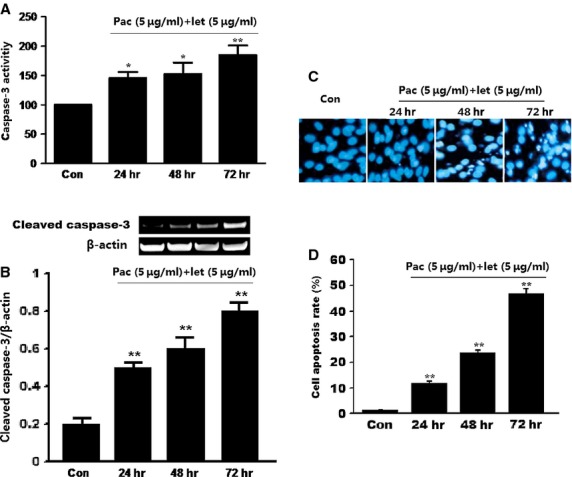
Effects of the combination of paclitaxel and letinan on the caspase-3 activities (A), the cleaved caspase-3 expression (B) and cell apoptosis (C and D) of A549 cell. Data represent the mean ± SEM, *P < 0.05, **P < 0.01 *versus* Con group. Con: control group; pac: paclitaxel; let: letinan.

Reactive oxygen species over-production associated with mitochondrial dysfunction induces the production of pro-apoptotic proteins, and subsequently causes caspase-dependent apoptosis. Thus, we measured the activity of caspase-3. As shown in Figure6A, after treatment with paclitaxel and letinan for 24, 48 and 72 hrs, caspase-3 activity of A549 cells was significantly increased to 1.46 times, 1.53 times and 1.85 times, respectively, compared with that in control group. We also found that co-treatment up-regulated the protein expression of cleaved caspase-3, which is the active form of caspase-3 (Fig.6B). These results suggested that combined treatment of the two drugs could promote the activation of caspase-3.

We also determined the effects of combined treatment on cell apoptosis *via* Hoechst33342 staining. We found significantly increased cell apoptosis in A549 cells with combined treatment of paclitaxel with letinan. As shown in Figure6C and D, cell apoptosis rate of control group was 1.22 ± 0.18%, but the apoptosis rate was time-dependently increased to 11.43 ± 1.26% (24 hrs), 23.45 ± 1.34% (48 hrs) and 46.55 ± 2.21% (72 hrs) in co-treated groups.

## Discussion

Paclitaxel is a commonly used chemotherapeutic drug for cancer. However, the failure of paclitaxel-based chemotherapy due to the drug resistance or the side effects limits its efficacy. Recently, some studies demonstrated that lentinan had many properties such as anti-tumour, immunological modulation and antioxidant 11,15,16. Beneficial effects have been gained in many lentinan-treated cancer patients. In this study, we found that letinan exerted synergistic effects with paclitaxel in promotion of A549 cell apoptosis as well as inhibition of cell proliferation. Our further study revealed that activations of ROS-TXNIP-NLRP3 inflammasome and ASK1/p38 MAPK signal pathways were involved in the synergistic anti-cancer effects.

Apoptosis is characterized by a series of biochemical and morphological changes. One of the most significant events in apoptosis is mitochondrial dysfunction and ROS over-production. The thioredoxin system, acting as an important antioxidant system, interacts with target molecules and exerts key biologic activities including growth control and anti-apoptotic properties 17–19. Thioredoxin can bind to signalling molecules such as ASK-1 and TXNIP that potentially influence cell growth and survival in diverse human diseases such as cancer, diabetes, and heart disease 19,20. The recent study by Zhou *et al*. identified TXNIP as a link between ROS and inflammasome activation 21. Reactive oxygen species has been proven to be critical for inflammasome activation in response to a number of stimuli. Reactive oxygen species induces the dissociation of TXNIP from thioredoxin and allows it to bind NLRP3. Consistently, we found that co-treatment with paclitaxel and letinan not only up-regulated TXNIP expression, but also enhanced TXNIP-NLRP3 interaction. Correspondingly, co-treatment induced the activation of NLRP3 inflammasome, and increased the expressions of cleaved caspase-1 and IL-1β. Therefore, our data suggest a model whereby co-treatment with paclitaxel and letinan results in TXNIP release from TRX because of ROS-induced oxidation, which allows TXNIP to interact with NLRP3 inflammasome. Finally, NLRP3 inflammasome activation promotes the processing of pro-ILβ to mature IL-1β and induces cell apoptosis.

In addition, accumulation of ROS has been verified to act as a second messenger to activate the ASK1/p38 MAPK pathway 22,23. Reactive oxygen species disrupts ASK1 association with thioredoxin, and induces ASK1 activation. Subsequently, ASK1 acts as an upstream mediator to activate p38 MAPK 24–26. In this study, co-treatment with paclitaxel and letinan increased intracellular ROS levels in a time dependent manner that induced activations of ASK1/p38 MAPK signal pathway. Our data were consistent with the evidence that p38 MAPK is a major downstream mediator of the ASK1-driven signalling pathways initiated by ROS. Consequently, ASK1 propagates apoptosis through the mitochondria-dependent caspase pathway. In our study, we found that co-treatment with paclitaxel and letinan significantly promoted caspase-3 activation thereby enhancing A549 cell apoptosis.

**Figure 7:**
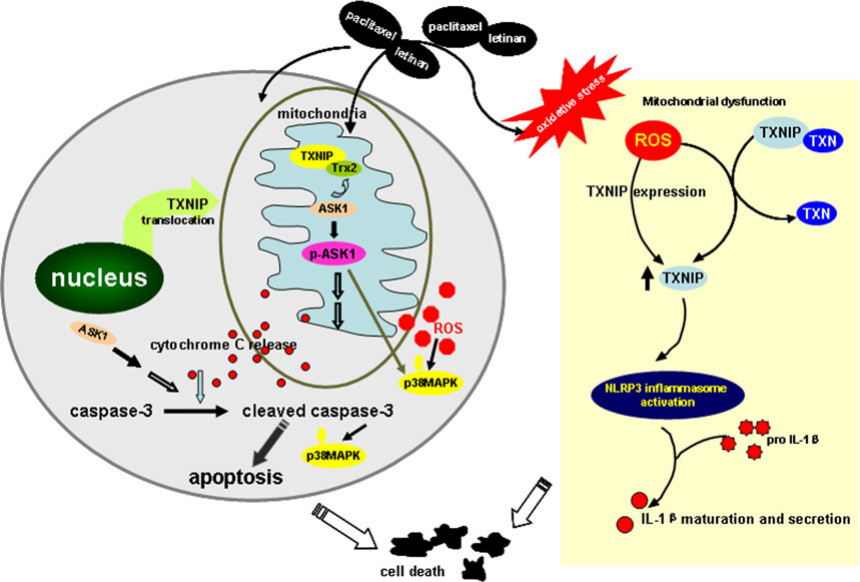
Schematic of the proposed mechanism involved in co-treatment with paclitaxel with letinan-induced cell apoptosis. Combination of paclitaxel and letinan induces ROS over-production, and activates NLRP3 inflammasome and ASK1/p38 MAPK signal, which are contributed to cell apoptosis. ROS: reactive oxygen species; ASK1: apoptosis signal regulating kinase-1; MAPK: mitogen-activated protein kinase; TRX: thioredoxin; TXNIP: thioredoxin-interacting protein.

In conclusion, co-treatment with paclitaxel and lentinan plays a synergistic role in enhancing A549 cell apoptosis. Activations of ROS-TXNIP-NLRP3 inflammasome and ASK1/p38 MAPK signal pathways contributed to these synergistic effects (as shown in Fig.7). Our results suggest that combined therapy with paclitaxel and lentinan may have a potential clinical perspective in cancer treatments.
